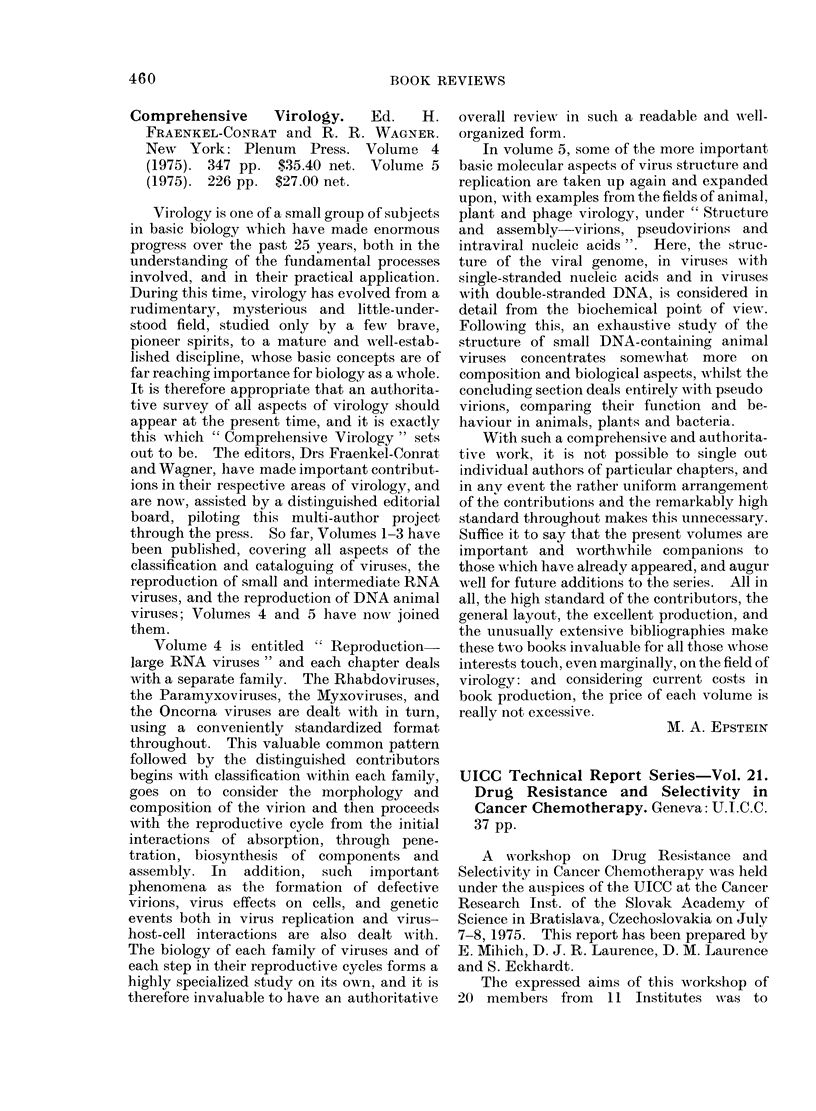# Comprehensive Virology

**Published:** 1976-10

**Authors:** M. A. Epstein


**Comprehensive Virology.** Ed. H. FRAENKEL-CONRAT and R. R. WAGNER. New York: Plenum Press. Volume 4 (1975). 347 pp. $35.40 net. Volume 5 (1975). 226 pp. $27.00 net.

Virology is one of a small group of subjects in basic biology which have made enormous progress over the past 25 years, both in the understanding of the fundamental processes involved, and in their practical application. During this time, virology has evolved from a rudimentary, mysterious and little-understood field, studied only by a few brave, pioneer spirits, to a mature and well-established discipline, whose basic concepts are of far reaching importance for biology as a whole. It is therefore appropriate that an authoritative survey of all aspects of virology should appear at the present time, and it is exactly this which " Comprehensive Virology " sets out to be. The editors, Drs Fraenkel-Conrat and Wagner, have made important contributions in their respective areas of virology, and are now, assisted by a distinguished editorial board, piloting this multi-author project through the press. So far, Volumes 1–3 have been published, covering all aspects of the classification and cataloguing of viruses, the reproduction of small and intermediate RNA viruses, and the reproduction of DNA animal viruses; Volumes 4 and 5 have now joined them.

Volume 4 is entitled " Reproduction—large RNA viruses " and each chapter deals with a separate family. The Rhabdoviruses, the Paramyxoviruses, the Myxoviruses, and the Oncorna viruses are dealt with in turn, using a conveniently standardized format throughout. This valuable common pattern followed by the distinguished contributors begins with classification within each family, goes on to consider the morphology and composition of the virion and then proceeds with the reproductive cycle from the initial interactions of absorption, through penetration, biosynthesis of components and assembly. In addition, such important phenomena as the formation of defective virions, virus effects on cells, and genetic events both in virus replication and virus–host-cell interactions are also dealt with. The biology of each family of viruses and of each step in their reproductive cycles forms a highly specialized study on its own, and it is therefore invaluable to have an authoritative overall review in such a readable and well-organized form.

In volume 5, some of the more important basic molecular aspects of virus structure and replication are taken up again and expanded upon, with examples from the fields of animal, plant and phage virology, under " Structure and assembly—virions, pseudovirions and intraviral nucleic acids ". Here, the structure of the viral genome, in viruses with single-stranded nucleic acids and in viruses with double-stranded DNA, is considered in detail from the biochemical point of view. Following this, an exhaustive study of the structure of small DNA-containing animal viruses concentrates somewhat more on composition and biological aspects, whilst the concluding section deals entirely with pseudo virions, comparing their function and behaviour in animals, plants and bacteria.

With such a comprehensive and authoritative work, it is not possible to single out individual authors of particular chapters, and in any event the rather uniform arrangement of the contributions and the remarkably high standard throughout makes this unnecessary. Suffice it to say that the present volumes are important and worthwhile companions to those which have already appeared, and augur well for future additions to the series. All in all, the high standard of the contributors, the general layout, the excellent production, and the unusually extensive bibliographies make these two books invaluable for all those whose interests touch, even marginally, on the field of virology: and considering current costs in book production, the price of each volume is really not excessive.

M. A. EPSTEIN